# Brugada Syndrome in Women: What Do We Know After 30 Years?

**DOI:** 10.3389/fcvm.2022.874992

**Published:** 2022-04-11

**Authors:** Estefanía Martínez-Barrios, Elena Arbelo, Sergi Cesar, José Cruzalegui, Victoria Fiol, Nuria Díez-Escuté, Clara Hernández, Ramon Brugada, Josep Brugada, Oscar Campuzano, Georgia Sarquella-Brugada

**Affiliations:** ^1^Arrhythmia, Inherited Cardiac Diseases and Sudden Death Unit, Hospital Sant Joan de Déu, University of Barcelona, Barcelona, Spain; ^2^Arrhythmia Section, Cardiology Department, Hospital Clínic, Universitat de Barcelona, Barcelona, Spain; ^3^Institut d’Investigació August Pi i Sunyer (IDIBAPS), Barcelona, Spain; ^4^Centro de Investigación Biomédica en Red de Enfermedades Cardiovasculares (CIBERCV), Madrid, Spain; ^5^Medical Science Department, School of Medicine, University of Girona, Girona, Spain; ^6^Cardiovascular Genetics Center, University of Girona-Institut d’Investigacions Biomèdiques de Girona (IDIBGI), Girona, Spain; ^7^Cardiology Service, Hospital Josep Trueta, University of Girona, Girona, Spain

**Keywords:** brugada syndrome, women, arrhythmias, sudden cardiac death, gender

## Abstract

Brugada syndrome (BrS) was initially described in 1992 by Josep and Pedro Brugada as an arrhythmogenic disease characterized by ST segment elevation in the right precordial leads and increased risk of sudden cardiac death (SCD). Alterations in the *SCN5A* gene are responsible for approximately 30% of cases of BrS, following an autosomal dominant pattern of inheritance. However, despite its autosomal transmission, sex-related differences are widely accepted. BrS is more prevalent in males than in females (8–10 times), with males having a 5.5-fold higher risk of SCD. There are also differences in clinical presentation, with females being more frequently asymptomatic and older than males at the time of diagnosis. Some factors have been identified that could explain these differences, among which testosterone seems to play an important role. However, only 30% of the available publications on the syndrome include sex-related information. Therefore, current findings on BrS are based on studies conducted mainly in male population, despite the wide acceptance of gender differences. The inclusion of complete clinical and demographic information in future publications would allow a better understanding of the phenotypic variability of BrS in different age and sex groups helping to improve the diagnosis, management and risk management of SCD.

## Introduction

**FIGURE 1:**
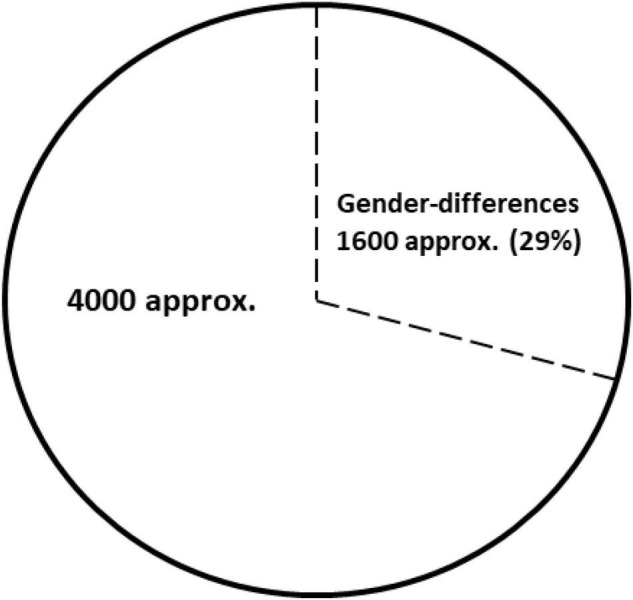
Publications focused on Brugada Syndrome (BrS) (PubMed, January 2022). Of approximate 5,600 publications about BrS near 29% (1,600 publications) included any data concerning female/women or gender/sex differences.

**FIGURE 2:**
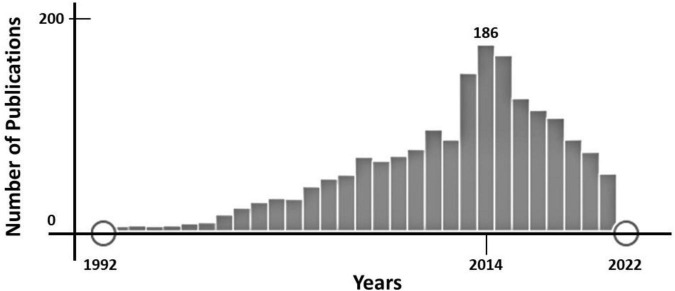
Time-line of publications focused on Brugada Syndrome (BrS) (PubMed, January 2022). Since 2014, the number of publications including any data concerning female/women or gender/sex differences has decreased progressively.

Thirty years ago, Josep and Pedro Brugada reported a new clinical entity characterized by “*Right bundle branch block, persistent ST segment elevation and sudden cardiac death*.” In this first report, two of eight patients described were females, suggesting potential gender differences (1). In 1996, Japanese researchers coined the term Brugada syndrome (BrS) when referring to this syndrome (2). Two years later, the first genetic alteration to cause this condition was reported in *SCN5A*, following an autosomal dominant pattern of inheritance. This gene encodes the α subunit of the cardiac sodium channel protein (Nav1.5) responsible for the initial upstroke of the action potential (3). Current guidelines define BrS as “a trait inherited in an autosomal dominant manner and showing sex- and age-related penetrance and variable expressivity.” Clinical manifestations are more common in adults, and eightfold more frequent in males than in females (4, 5). Therefore, 30 years after the first description of BrS, gender differences are widely accepted, but its underlying causality remains unclear and further research is needed. To date, only about 1,600 (approx. 29%) of around 5,600 papers focused on BrS (PubMed, January 2022) include any data concerning female/women or gender/sex differences (Figure 1). It is also important to remark that, despite the extensively accepted differences between genders and the increasing number of publications up to 2014, publications including any data regarding gender differences has progressively decreased in recent years (Figure 2).

## Clinical Findings

In 1992, the first report of BrS included six males and two females, suggesting potential gender differences despite the low number of cases and inclusion of infants (1). In 1997, nearly fifty patients were reported worldwide to have BrS (only three were women) (6). Two years later, the number of reported BrS patients increased to one hundred and 60 (13 were female) (7). At that time, gender differences were widely accepted in BrS, but no explanation was reported.

After ten years, BrS was phenotypically and genetically known as sudden unexpected death syndrome (SUDS), known for many years in southern Asia and characterized by a disproportionate number of men died suddenly, usually sleeping (8). Also in 2002, the first BrS consensus was published, focusing on diagnostic criteria and reporting a male predominance (8:1 ratio) (9). At the clinical level, males showed easier inducibility of BrS pattern on ECG and a higher number of events on follow-up compared to females (10, 11). In 2005, the second consensus conference was published, and stated that male sex was a 5.5-fold greater risk factor for SCD than female sex, although no data concerning the cellular mechanisms involved were included, mainly due to lack of conclusive mechanistic/physiopathologic evidence (12). In 2008, a study reported that women with the BrS resuscitated from cardiac arrest or with appropriate ICD shocks exhibit a different ECG pattern than men, suggesting that it may be more difficult to identify women with BrS who are at risk for SCD (13).

In 2013 HRS/EHRA/APHRS expert consensus statement declared that “*BrS is 8-10 times more prevalent in men than in women*” and “*Male sex has consistently been shown to be associated with more arrhythmic events*” (14). However, no further reference to gender differences was mentioned. In 2015, ESC/AEPC guidelines for the management of patients with ventricular arrhythmias and the prevention of SCD were published (4). BrS was listed but no reference to gender differences were included, despite mention of male predominance. In 2017, J Wave Syndrome Consensus Conference report stated a male predominance in BrS, potentially due to “*Testosterone modulation of ion currents underlying the epicardial AP notch*” (15). No other reference to gender differences in BrS was mentioned. Similar data concerning gender differences were included in AHA/ACC/HRS Guidelines (5). In 2018, the Shanghai Score System was proposed focused on diagnosis and risk stratification of BrS patients but, the cohort included more than 90% men, probably as a result of the male predominance in BrS. No inclusion of any additional data concerning gender differences was reported, despite its wide acceptance (16).

Hence, although few data concerning clinical translation of BrS gender differences published so far, it is accepted that women with BrS are more frequently asymptomatic at the time of diagnosis and older than men both at the time of diagnosis and with the first arrhythmic event (17, 18). In addition, women with BrS show a spontaneous type 1 Brugada ECG pattern or ventricular arrhythmia inducibility less frequently than men (19, 20). Furthermore, women diagnosed with BrS are less likely to experience arrhythmic events (syncope, aborted cardiac arrest, and documented ventricular fibrillation) (18, 19). Following similar data, BrS ECG patterns are not uncommon in elderly women, but are not associated with an increased risk of mortality (21, 22). Recently, a new study demonstrated that women with BrS less frequently presented with a type 1 ECG pattern, had a higher rate of family history of SCD, and had less sustained ventricular arrhythmias on electrophysiological study, despite not constituting a risk-free group. Concerning the risk of malignant events, only atrial fibrillation and positive genetic test were found as risk factors for further arrhythmic events. Neither clinical risk factors nor electrophysiological study predicts future arrhythmic episodes in women, making correct risk stratification difficult (23).

## Age Differences in Women

The aforementioned data were performed on young-adult and adults women diagnosed with BrS. Regarding early years, few studies contain diagnosed children, despite the first report already included two pediatric-aged female (1). In large cohorts of asymptomatic children, the characteristic ECG pattern was identified in 0.01–0.02%; it suggests that BrS exist in children but becomes clinically unmasked with increasing age (24, 25). The incidence rate of life-threatening arrhythmias in the pediatric population was showed to be around 10%, with fever as trigger for ventricular arrhythmias (26, 27). In addition, there is at most a mild male predominance of BrS in the pediatric population compared to adults. And women show a higher rate of arrhythmic events in the pediatric age group than at an older age (17). Curiously, almost 25% of asymptomatic children who were first-degree relatives of BrS patients showed characteristic BrS ECG on ajmaline test after puberty, despite showing normal ECG also on ajmaline test before adolescence (28); it reinforces the role of hormones in BrS (29). Therefore, pediatric cases are rare and are usually identified during familial screening, but children often have a more severe form of the disease, which manifests as a quickly progressive manner and lead to malignant arrhythmias and SCD (26, 30–32). Patients showing an ECG type I and a history of syncope or aborted SCD should receive an ICD implantation (class I indication). Contrarywise, ICD implantation is not indicated in asymptomatic patients without risk factors (33).

Concerning elderly BrS patients, scattered data have been published to date, showing that BrS ECG patterns are less frequent than in adults, with similar episodes in both genders and a reduced risk of life-threatening arrhythmias (22). There is no strong evidence that levels of testosterone decrease during aging, thus/thereby reducing the risk of malignant events. Although decreased testosterone levels are associated with comorbidities, it is important to remark that the treatment of these comorbidities includes many drugs that should be avoided in BrS^1^ (34). The device-guided management should be personalized. A personalized approach should be done before ICD implantation. At our acknowledgement, the first and only study focused on elderly BrS women was published in 2020, showing a not infrequent BrS pattern in the ECG but associated with a lower risk of malignant arrhythmias and SCD (21).

## Pregnancy

Following the lack of data on BrS in women, few studies focusing on pregnancy in BrS diagnosed women have been published to date. First studies emphasized the role of hormonal changes during pregnancy as trigger for arrhythmic events (35) but typical ECG changes of BrS may be linked to sodium channel blockers used as anesthetics (36). The first large serie was published in 2014, showing that serious events were not more frequent during pregnancy or the peripartum period (37). Finally, women with BrS might have an overall low tendency to malignant arrhythmias during pregnancy (38) and obstetrical management should include a multidisciplinary follow-up carried out in a close collaboration between gynecologists, pediatricians, cardiologists and anesthesiologists.

## Cellular Basis

Since the first report in 1992, gender differences were widely accepted in BrS, nevertheless no explanation was published in 2002. Di Diego et al. demonstrated a more prominent transient outward current (Ito) in males than in females in right ventricular epicardium of dogs (39). Therefore, gender differences in BrS due to intrinsic differences in the ventricular action potential between genders were suggested. One year on, in 2003, sex hormones were also proposed as another factor contributing to the male predominance in BrS. Especially testosterone that may accentuate ST-segment elevation by increasing outward currents (Ito, IKr, IK1…) or decreasing inward currents (ICa-L, INa…) at the end of phase 1 of the action potential (40, 41). In 2005, a potential role for gonadal steroids in gender-related differences in cardiac repolarization and BrS susceptibility was suggested (42, 43). In 2007, Shimizu et al. reported higher testosterone levels, serum sodium, potassium and chloride levels, as well as a significantly lower body-mass index in males diagnosed with BrS (44). In the same year, Eckardt reviewed all published studies concerning patients with BrS (more than 1,200 up to 2.006) and observed that 80% were males. Authors suggested that gonadal steroids seem to be an unlikely single explanation for gender differences in BrS. Therefore, BrS differences may be due to a complex interaction between gender- and age-dependent genetic and other triggering and/or modulating factors such as circadian variations of vagal balance, hormones, and metabolic factors, among others (45, 46).

Focusing on mechanistic pathways, it is currently accepted that transmural voltage gradient created by an imbalance in the cardiac ion currents involved in phase 1 of the action potential is the cause of the typical Brugada-type ST segment elevation observed mainly in men; it is due to a loss of function of the sodium or calcium inward depolarization current and a gain of function of the transient outward potassium current (Ito) (47). Ito is higher in males and may facilitate the presence of the BrS ECG pattern and arrhythmias. In addition, testosterone may increase outward repolarizing currents, leading to loss of the AP dome (48). In line with this hypothesis, the delayed right ventricular ejection, more frequently observed in males than in females, could contribute to an increase risk of malignant events in BrS (49). In concordance to this fact, in 2019 a case report of a female living as a transgender male was reported, in which testosterone supplementation unmasked the BrS ECG pattern (50).

## Genetics

In 1998, the first genetic alteration associated with BrS was reported, confirming genetic basis as cause of BrS already suggested in 1992 (1). The first genetic alteration was reported in *SCN5A*, following an autosomal dominant pattern of inheritance. Then, two hallmarks of BrS were identified: incomplete penetrance and variable expressivity. Pathogenic alterations in this gene leads to loss of function in the α subunit of the cardiac sodium channel protein (Nav1.5). To date, more than 150 deleterious alterations in *SCN5A* have been associated with BrS and underlie nearly 30% of all BrS cases (49, 51). Although several genetic alterations located in more than 20 genes have been reported as potentially cause of BrS (52) recent evidence-based reappraisal of gene-disease validity disputed the causality of main part of these genes, leaving *SCN5A* as the only gene with definite causality in BrS (53). In addition, a recent study suggested few minor genes as high potential cause of BrS (*SLMAP*, *SEMA3A*, *SCNN1A*, and *SCN2B*) (54). Due to low genetic yield after a comprehensive genetic analysis, other patterns of inheritance have been also suggested for BrS families (51). Nowadays, it is widely accepted an 8–10-fold male BrS predominance despite equal genetic transmission. Hence, carriers of a deleterious variant in the *SCN5A* gene showed more aggressive arrhythmias (55). However, in recent years a higher prevalence of pathogenic variants in *SCN5A* has been published in asymptomatic female patients with BrS compared with male patients and an even high prevalence in female patients with BrS with arrhythmic events (20) suggesting that female patients carrying a pathogenic variant in *SCN5A*, may be a marker of increased risk (56).

## Conclusion

Nowadays, the existence of sex-attributable differences in the prevalence, risk profile and clinical course of BrS is widely accepted. Current knowledge supports that such differences are not exclusively due to the influence of sex hormones, but may be the result of a complex interplay of gender- and age-dependent genetic factors and other variables that modulate the expression and function of cardiac ion channels. However, further studies are still needed to elucidate the pathophysiological mechanisms underlying these gender differences. In general, women have a lower prevalence of BrS, a lower risk of arrhythmic events, and are more frequently asymptomatic and older at the time of diagnosis than their male counterparts. Despite this, the female sex does not represent a risk-free group and the fact that they present less frequently with the ECG BrS pattern in the electrophysiological study could hinder its diagnosis. Nevertheless, current expert guidelines on the management and risk stratification of BrS patients do not differ in their recommendations according to sex, probably due to the low number of published data on female patients. Although current studies in young, pregnant and menopausal women with BrS predict a low risk of events and lethality, data are scarce. More in-depth evaluation of the influence of female hormonal changes on the BrS phenotype, as well as the cellular mechanisms involved, is needed. We recommend including as complete as possible clinical and phenotypic information on BrS patients in future publications. A more detailed knowledge of the course of the syndrome in different age and sex groups would allow adapting clinical recommendations toward individualized care in the diagnosis, management and risk stratification of women with BrS.

## Author Contributions

GS-B, OC, EA, JB, and RB developed the concept and supervised the study. EM-B, EA, SC, JC, VF, ND-E, CH, and GS-B acquired, pre-processed, and analyzed the data. EM-B, EA, OC, and GS-B prepared the manuscript. All authors contributed to manuscript revision, read and approved the submitted version.

## Conflict of Interest

The authors declare that the research was conducted in the absence of any commercial or financial relationships that could be construed as a potential conflict of interest.

## Publisher’s Note

All claims expressed in this article are solely those of the authors and do not necessarily represent those of their affiliated organizations, or those of the publisher, the editors and the reviewers. Any product that may be evaluated in this article, or claim that may be made by its manufacturer, is not guaranteed or endorsed by the publisher.

## Footnotes

1
www.BrugadaDrugs.org
